# The in vitro addition of idarucizumab to plasma samples from patients increases thrombin generation

**DOI:** 10.1038/s41598-021-85318-y

**Published:** 2021-03-15

**Authors:** Mojca Božič Mijovski, Rickard E. Malmström, Nina Vene, Jovan P. Antovic, Alenka Mavri

**Affiliations:** 1 Department of Vascular Diseases, University Medical Centre Ljubljana, Zaloška c. 7, 1000 Ljubljana, Slovenia; 2 Department of Medicine Solna, Karolinska Institutet and Clinical Pharmacology, Karolinska University Hospital, Stockholm, Sweden; 3 Department of Coagulation Research, Institute for Molecular Medicine and Surgery, Karolinska Institutet, and Department of Clinical Chemistry, Karolinska University Hospital, Stockholm, Sweden; 4 Division of Internal Medicine, Faculty of Medicine, University of Ljubljana, Ljubljana, Slovenia; 5 Faculty of Pharmacy, University of Ljubljana, Ljubljana, Slovenia

**Keywords:** Translational research, Cardiology

## Abstract

Dabigatran interferes with many coagulation tests. To overcome this obstacle the use of idarucizumab as an in vitro antidote to dabigatran has been proposed. The aim of this study was to test the effect of idarucizumab as an in vitro antidote to dabigatran in ex vivo plasma samples from routine clinical patients examined by a thrombin generation assay (TGA). From 44 patients with atrial fibrillation five blood samples were collected. Thrombin generation was measured in all samples before and after the addition of idarucizumab. When idarucizumab was added to baseline plasma (no dabigatran), it caused a significantly shorter Lag Time and Time to Peak Thrombin, and a higher Peak Thrombin and Endogenous Thrombin Potential (ETP) of TGA. Similar results were obtained when idarucizumab was added to dabigatran-containing plasma, with TGA parameters comparable to baseline + idarucizumab plasma, but not to baseline plasma. In summary, our study showed that in vitro addition of idarucizumab to plasma samples from patients increases thrombin generation. The use of idarucizumab to neutralize dabigatran in patient plasma samples as well as the clinical relevance of in vitro increased thrombin generation induced by idarucizumab needs further investigation.

## Introduction

Dabigatran etexilate is an oral prodrug that is converted into its active compound, dabigatran, a direct thrombin inhibitor that acts on both clot-bound and free thrombin^1^. Dabigatran has been clinically developed for the prevention and treatment of thromboembolic events in patients with atrial fibrillation or venous thromboembolic disease. It has several advantages over vitamin K antagonists (VKA), such as no need for continuous laboratory monitoring, limited drug-drug interactions, no dietary interactions, fixed-dose regimen, predictable anticoagulant activity, etc.^2^. One of the disadvantages of using dabigatran had been the lack of a specific antidote. This disadvantage has been overcome by the introduction of idarucizumab (Praxbind, Boehringer Ingelheim), a humanized monoclonal antibody Fab fragment that binds unbound and thrombin-bound dabigatran and its active glucuronide metabolites and forms stoichiometric 1:1 complexes. The affinity of dabigatran for idarucizumab is ≈350 times higher than the affinity of dabigatran for thrombin. Once dabigatran is complexed with idarucizumab, the anticoagulant effects of unbound and protein-bound dabigatran and its active metabolites are rapidly neutralized^3^. Idarucizumab is specific for dabigatran and has no known endogenous targets^4^. In animals and human volunteers, the administration of idarucizumab did not cause any obvious procoagulant effects^3,5^. Idarucizumab also quickly and completely reversed anticoagulation in patients treated with dabigatran, who had uncontrolled bleeding or underwent urgent procedures, and no explicit thrombotic events associated with idarucizumab have been reported^6^.

In patients receiving dabigatran therapy, thrombophilia testing or a coagulation test may be required to diagnose a hemostatic disorder such as vitamin K deficiency, liver disease, or acquired haemophilia A. Such testing is not possible during therapy because dabigatran interferes with many coagulation tests and leads to false positive or false negative results^7–10^. To overcome this obstacle and facilitate diagnosis, the use of idarucizumab as an in vitro antidote to dabigatran has been suggested^11^. However, this procedure has so far only been tested on pool plasma spiked with dabigatran. Furthermore, only a limited battery of tests was used. Coagulation tests such as prothrombin time (PT), activated partial thromboplastin time (APTT) and thrombin time (TT) measure the time it takes for clotting to start, when only a tiny fraction of thrombin has been formed (< 2%) and therefore provide only partial information about the haemostatic system. In contrast, thrombin generation assay (TGA) measures thrombin activity that develops in a clot and is proportional to the bleeding or thrombotic tendency^12^. Therefore, this study investigated the effect of idarucizumab as an in vitro dabigatran antidote in ex vivo plasma samples from patients in routine clinical practise using the TGA.

## Results

The addition of saline to plasma had no effect on Lag Time, Time to Peak Thrombin (TPT) and Peak Thrombin, but increased Endogenous Thrombin Potential (ETP) by 3% on average (Supplementary Table 1). Screening coagulation times were also not affected.

The effect of the in vitro addition of idarucizumab was initially tested in baseline plasma samples. Idarucizumab significantly shortened Lag Time and TPT, while Peak Thrombin and ETP were increased by 16% and 14%, respectively. PT (%) and APTT ratio significantly increased, while idarucizumab had no effect on TT (Table 1).

**Table 1:** TGA and screening coagulation tests before and after the addition of idarucizumab to either plasma without (baseline and baseline + I) or with dabigatran. Average ± SD and paired t-test p values are shown.

	Baseline (n = 44)	Baseline + I (n = 44)	p*	DABI	DABI + I	p^#^
**Thrombin generation**						
Lag time (min)	17.3 ± 3.3	15.9 ± 3.1	< 0.001	37.8 ± 10.9	15.9 ± 4.2	< 0.001
Peak Thrombin (nM)	202 ± 87	235 ± 92	< 0.001	194 ± 113	271 ± 94	< 0.001
TPT (min)	24.6 ± 4.7	23.0 ± 4.3	< 0.001	44.0 ± 10.8	22.3 ± 5.5	< 0.001
ETP (nM x min)	3390 ± 738	3860 ± 873	< 0.001	2281 ± 1179	4138 ± 702	< 0.001
**Screening coagulation tests**						
PT (%)	91 ± 9	96 ± 12	= 0.002	0.66 ± 0.12	0.94 ± 0.17	< 0.001
APTT (ratio)	0.98 ± 0.08	1.05 ± 0.09	< 0.001	1.83 ± 0.44	1.03 ± 0.13	< 0.001
TT (s)	16.8 ± 1.3	17.0 ± 1.8	NS	–	16.6 ± 0.8	NA

*APTT* Activated Partial Thromboplastin Time, *ETP* Endogenous Thrombin Potential, *I* idarucizumab, *NS* not significant, *PT* Prothrombin Time, *TGA* Thrombin Generation Assay, *TPT* Time to Peak Thrombin.

*Paired t-test between baseline and baseline + I.

^#^Paired t-test between DABI and DABI + I.

**Figure 1:**
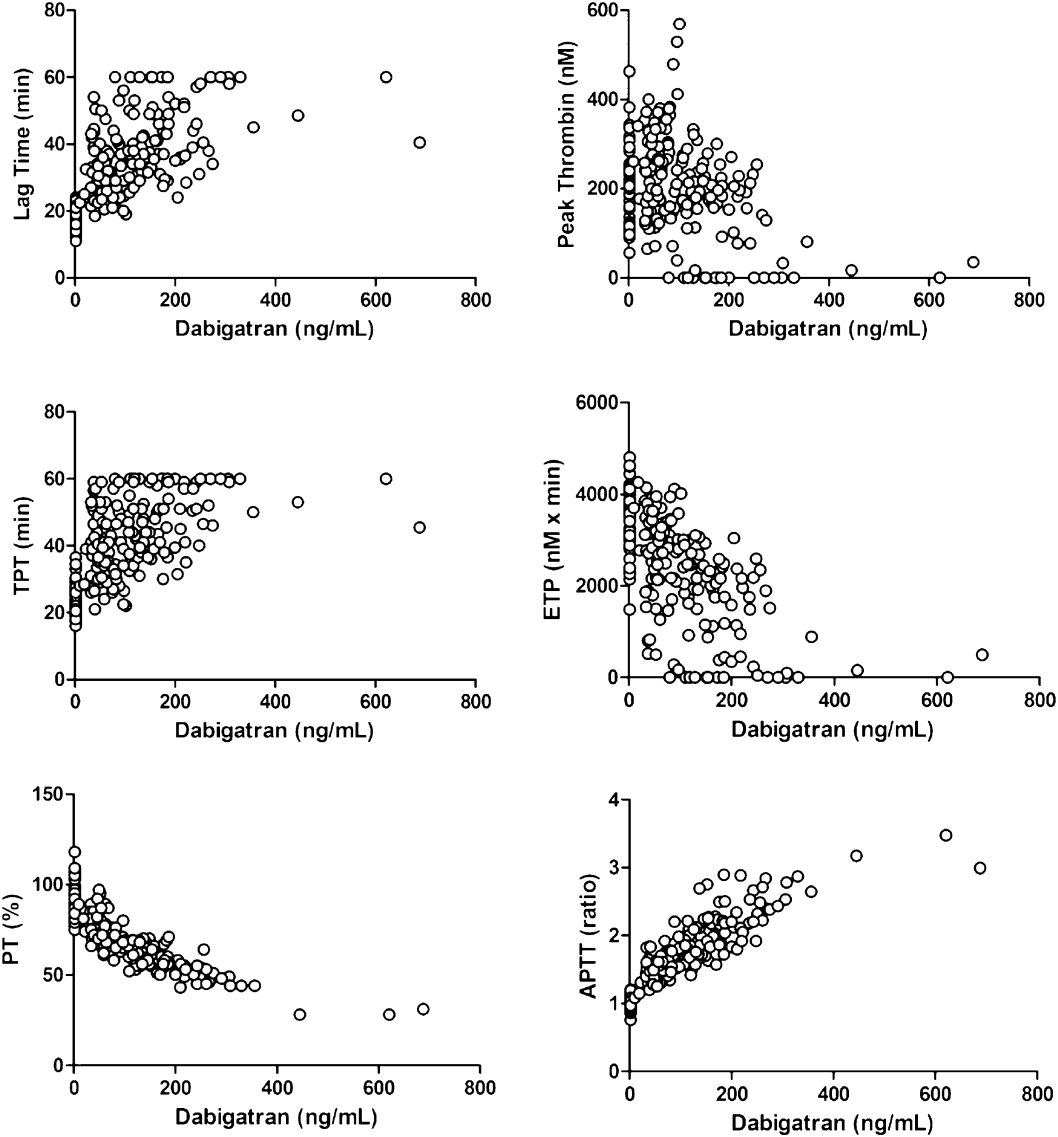
Correlations between dabigatran concentration and TGA (Lag Time, Peak Thrombin, TPT and ETP), PT and APTT. *TGA* Thrombin Generation Assay, *TPT* time to peak thrombin, *ETP* Endogenous Thrombin Potential, *PT* Prothrombin Time, *APTT* Activated Partial Thromboplastin Time.

In ex vivo plasma samples (DABI) dabigatran concentration ranged from 10 to 688 ng/mL being on average (± SD) 79 ± 59 ng/mL at trough and on average 172 ± 105 ng/mL at peak. Dabigatran concentration correlated positively with TGA parameters Lag Time (r = 0.619) and TPT (r = 0.585), and negatively with Peak Thrombin (r = − 0.358), and ETP (r = − 0.618, all p < 0.001). Negative correlation was also observed for PT (%) (r = -0.827) and positive correlation for APTT ratio (r = 0.871 both p < 0.001) (Fig. 1).

**Figure 2:**
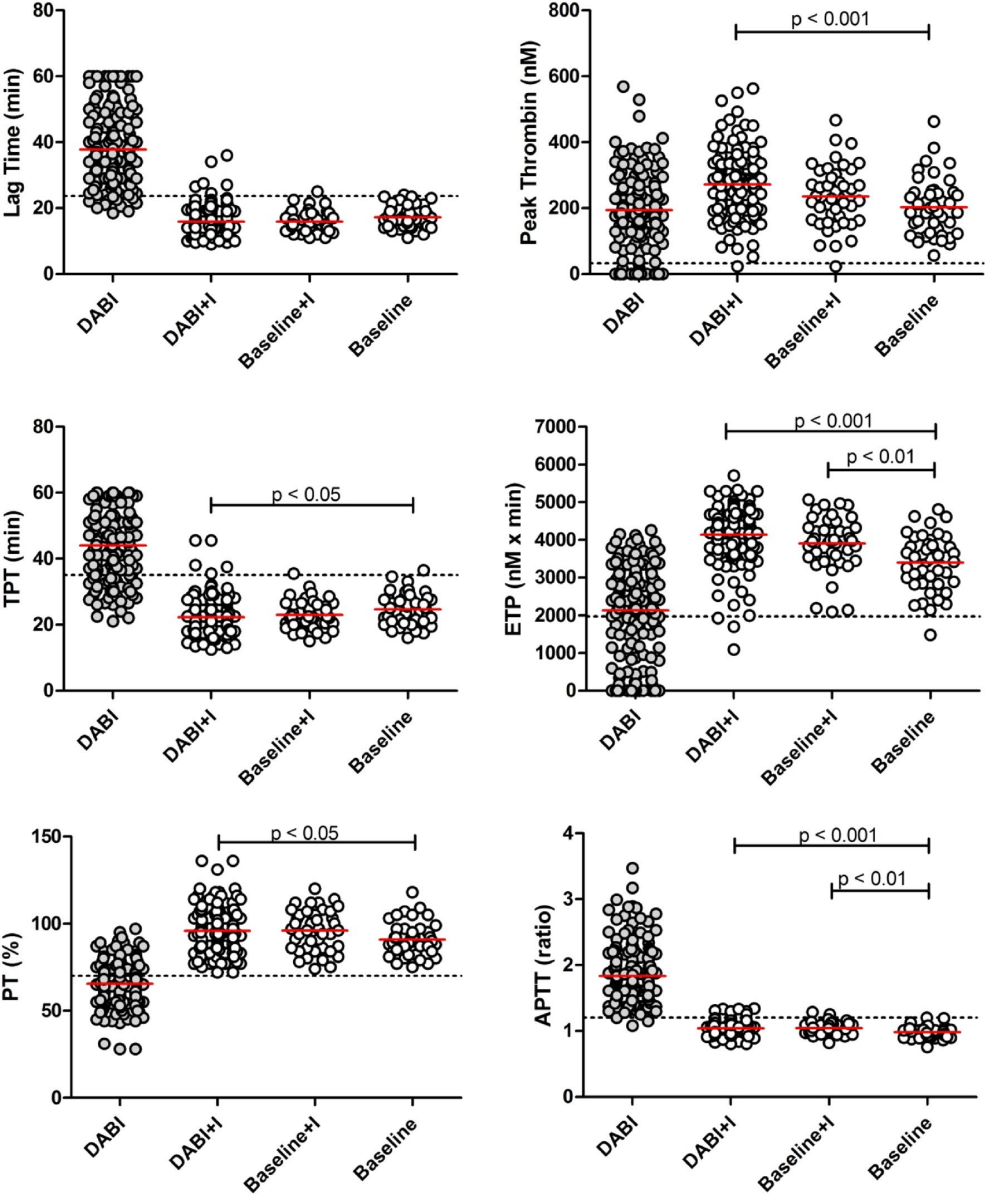
TGA (Lag Time, Peak Thrombin, TPT and ETP), PT and APTT are shown in baseline, baseline + I and DABI + I plasma. Dotted lines represent the lower (Peak Thrombin, ETP and PT) or the upper (Lag Time, TPT and APTT) reference values. Significant Tukey's multiple comparison post-hoc test p values for DABI + I, baseline + I and baseline are shown. *TGA* Thrombin Generation Assay, *TPT* Time to Peak Thrombin, *ETP* Endogenous Thrombin Potential, *PT* Prothrombin Time, APTT Activated Partial Thromboplastin Time, *Baseline* samples without dabigatran, *Baseline* + *I* samples without dabigatran spiked with idarucizumab, *DABI* + *I* samples with dabigatran spiked with idarucizumab.

The addition of idarucizumab to DABI plasma samples (DABI + I) significantly shortened Lag Time and TPT and increased Peak Thrombin and ETP (Table 1). No difference in any parameter was noted between DABI + I samples obtained at trough or at peak, therefore, neutralization of dabigatran with idarucizumab was equally effective throughout the whole range of dabigatran concentrations. All PT and TT results for DABI + I plasma were within the reference range, while several APTT ratios remained prolonged above the upper reference value (Fig. 2).

Coagulation test results in DABI + I plasma were, however, on average not comparable to that of baseline plasma samples, but rather to baseline + I plasma samples. When DABI + I test results were compared to baseline plasma samples, Peak Thrombin and ETP were significantly higher, while TPT was significantly shorter (Fig. 2). No significant difference was noted between DABI + I and baseline + I samples. PT (%) and APTT ratio were significantly higher in DABI + I plasma compared to baseline.

## Discussion

This study showed that the in vitro addition of idarucizumab to patient plasma samples increases thrombin generation, manifested as shorter TGA Lag Time and TPT together with increased peak thrombin and ETP. These effects were similar when idarucizumab was added to baseline (no dabigatran) plasma samples and when idarucizumab was added to ex vivo samples from patients under dabigatran therapy.

The effects of the in vitro addition of idarucizumab have been described by Jacquemin and colleagues^11^. In their study, normal pool plasma and pool plasma from patients with activated protein C resistance were used, in which the addition of idarucizumab had no effect as measured by screening coagulation (PT, APTT and TT) and factor (FVII, FVIII, FIX and FX) tests (normal pool plasma) or dilute Russel viper venom time (activated protein C resistance pool plasma). In a study by Bloemen and colleagues^13^, idarucizumab had no effect on TGA when added to one aliquot of normal pool plasma. Our laboratory confirmed the absence of a significant effect of idarucizumab when added to a single aliquot of normal pool plasma (results not shown), most likely due to the small number of test results (triplicate measurements) . However, when we tested a much larger number of patient samples, idarucizumab caused a significant shortening of TGA Lag Time and TPT and a significantly higher Peak Thrombin and ETP, which implies increased thrombin generation. These effects could not be attributed to the dilution caused by the addition of idarucizumab to plasma, which not only dilutes the procoagulant but also the anticoagulant factors (tissue factor pathway inhibitor, protein C, protein S, antithrombin), as dilution of plasma with saline in the same ratio as idarucizumab caused a 3% increase in ETP (compared to a 14% increase in ETP with idarucizumab) but had no significant effect on other TGA parameter or screening coagulation assays (Supplementary Table 1).

Results of the screening coagulation tests were inconsistent. PT (%) was significantly higher after addition of idarucizumab and was therefore consistent with the TGA results. On the contrary, significantly higher APTT ratio suggested idarucizumab may interfere with the intrinsic pathway of the coagulation cascade. The apparently discrepant TGA and APTT results can be explained by the concentration of tissue factor (5 pM) used in our experiment. At a low tissue factor concentration (e.g., 1 pM), TGA is affected by all the coagulation factors. With increasing concentration of tissue factor the influence of factors VIII, IX and XI on TGA diminishes. Low tissue factor concentration is commonly used to study the coagulation process in haemorrhagic diseases^14^. With the higher tissue factor concentration used in our study, TGA was less sensitive to coagulation factors of the intrinsic pathway, which influenced APTT designed to reflect the intrinsic coagulation pathway. TT, which only detects disturbances in fibrin polymerization or the presence of the thrombin inhibitor, was not affected by the addition of idarucizumab.

Dabigatran concentration correlated positively with TGA parameters Lag Time and TPT, and negatively with Peak Thrombin and ETP. Prolongation of Lag Time and TPT dependant on dabigatran concentration has already been reported by several studies, but these studies also reported a paradoxical increase of Peak Thrombin and ETP in samples containing dabigatran^13,15^. In all these studies, calibrated automated thrombinography (CAT) developed by Hemker et al*.* was used^16^. In the CAT assay, each sample is calibrated with a calibrator that is influenced by dabigatran, which leads to a subsequent overestimation of thrombin activity. In our study, a TGA was used that generates a separate calibration curve for each substrate batch, so no calibrator is added to the samples. Secondly, CAT uses a correction algorithm in which the fluorescence generated by α2-macroglobulin-thrombin activity is subtracted from the total thrombin activity, however, α2-macroglobulin-thrombin is inhibited by dabigatran to varying degrees during the TG measurement. The TGA used in our study does not account for the presence of the α2-macroglobulin-thrombin complex^17^. It appears that this type of TGA better demonstrates hemostatic status when a small thrombin inhibitor such as dabigatran is present in a sample.

As expected, all TGA parameters were restored after the addition of idarucizumab to dabigatran-containing plasma samples (DABI + I). However, the results of the DABI + I plasma samples were not comparable to baseline plasma samples (no dabigatran), but rather to baseline plasma spiked with idarucizumab (baseline + I). Therefore, idarucizumab increased thrombin generation in both baseline plasma samples and DABI plasma samples. The concentration of idarucizumab used in our experiments was chosen according to the previously published study in which the addition of 125 µg/mL idarucizumab fully neutralized dabigatran in concentrations up to 1500 ng/mL as measured with the TT^11^. We did not have enough sample aliquots to test different idarucizumab concentrations in ex vivo samples. From the TT results, which were all within the reference range, we can conclude that dabigatran was successfully neutralized. Whether lower idarucizumab concentrations could be equally effective without increasing thrombin generation remains to be determined. Although promising, we believe that the idea of in vitro neutralization of dabigatran with idarucizumab needs further investigation and may currently lead to the inaccurate diagnosis of hemostasis disorders in patients receiving dabigatran. Whether the increased thrombin generation caused by idarucizumab as demonstrated our study leads to clinically relevant events, needs to be clarified in clinical trials.

The main limitations of our study were firstly, that a single concentration of idarucizumab was tested, and secondly, that only one type of TGA and one tissue factor concentration were used to detect increased thrombin generation. As all samples had been used, it was not possible to perform different TGA with different tissue factor concentrations.

In conclusion, our study showed that the in vitro addition of idarucizumab to plasma samples from patients increases thrombin generation either when added to baseline (no dabigatran) plasma or when added to dabigatran-containing plasma. Increased thrombin generation could be detected as shorter Lag Time and TPT, and higher Peak Thrombin and ETP with a TGA not prone to dabigatran artefacts. The use of idarucizumab as an in vitro antidote for dabigatran needs further investigation. Whether the increased thrombin generation caused by idarucizumab, as demonstrated in our study, leads to clinically relevant events, will have to be clarified in future clinical trials.

## Patients and methods

The patient characteristics are described in detail elsewhere^18^. In short, 44 patients with atrial fibrillation who had started treatment with dabigatran at the Anticoagulation Clinic (University Medical Centre, Ljubljana, Slovenia) (23 patients on dabigatran 150 mg and 21 patients on dabigatran 110 mg twice daily) were admitted after giving their informed consent. The study was approved by the Medical Ethical Committee of the Slovenian Ministry of Health. All experiments were performed in accordance with relevant guidelines and regulations.

Blood was collected five times from all patients: at baseline (before the start of therapy, n = 44) and then twice at the trough (12 ± 1 h after the last dabigatran dose) and twice at the peak (120 ± 5 min after the last dabigatran dose), 2–4 weeks and 6–8 weeks after the baseline sampling (DABI samples). Three patients missed one of the appointments and 15 plasma aliquots were used for other purposes. Therefore, 155 DABI plasma samples were available for this study. Eight patients who did not receive anticoagulant therapy were included to test the dilution effect of idarucizumab.

Blood was collected from the antecubital vein with a 21-gauge needle in 4.5 mL vacuum tubes containing 0.11 mol/L sodium citrate (Becton Dickinson, Vacutainer System Europe, Germany). Platelet-poor plasma was prepared according to standard procedures^19^, aliquoted, snap frozen in liquid nitrogen and stored at – 75 °C until analysis.

One vial of idarucizumab (Praxbind, Boehringer-Ingelheim, Germany, 50 mg/mL) was diluted 1:4 with saline to obtain a 12.5 mg/mL solution, which was aliquoted and frozen. Prior to analysis, an aliquot of this solution was thawed and further diluted 1:10 with saline solution. This solution was added to plasma samples (baseline and DABI) in a volume ratio of 1:10. In most cases 50 µL of idarucizumab solution was added to 450 µL of plasma. If less plasma was available 40 µL of idarucizumab solution was added to 360 µL of plasma or 30 µL of idarucizumab solution was added to 270 µL of plasma, therefore always maintaining the 1:10 volume ratio of added idarucizumab and assuring the same final idarucizmab concentration (125 µg/mL) in all plasma samples (baseline + I and DABI + I samples, respectively). To test the dilution effect of idarucizumab, eight plasma samples from patients who did not receive anticoagulant therapy were treated in the same manner as described above, but saline was added instead of idarucizumab in the same ratio as in patient samples (1 volume of saline + 9 volumes of plasma, 50 µL of saline was added to 450 µL of plasma).

One plasma aliquote of baseline and DABI plasma was used for the measurement of dabigatran concentration utilizing LC–MS/MS^7^. Dabigatran was purchased from Alsachim (Strasbourg, France) and dabigatran-d3 (internal standard) from Toronto Research Chemicals (Ontario, Canada). 150 μL acetonitrile containing internal standard was added to 50 μL plasma. After shaking and centrifugation, a further two-fold dilution with mobile phase A (see below) was performed, after which the sample was gently shaken and re-centrifuged. Three μL of the final extract was injected into the LC–MS/MS system. Chromatographic separation of the analytes was achieved on an Acquity UPLC BEH column (Shield RP18, 2.1 × 50 mm, 1.7 μm) using a gradient run with mobile phase A (10 mM ammonium formate pH 4.5) and mobile phase B (0.1% formic acid in acetonitrile). The analytes were detected using a Waters Quattro Premier XE mass spectrometer operating in positive electrospray ionization (ESI) mode, utilizing selected reaction monitoring (SRM) with ion transitions 472 → 289 m/z for dabigatran and 475 → 292 m/z for the internal standard. The total analysis time was 3 min. The calibration curve for dabigatran in plasma was linear over the range 1.1 – 412 ng/mL. Samples above this range were diluted with normal pooled plasma for accurate quantitation. The limit of detection (LOD) was estimated to < 0.5 ng/mL. No interfering peaks were observed in 18 blank plasma samples at the retention times of dabigatran or the internal standard. Post-column addition of analytes (dabigatran and internal standard) was used to study ion suppression effects from plasma. No significant ion suppression occurred at the retention time of the analyte. Validation experiments with three levels of control samples (8.1, 202 and 393 ng/mL) on three different occasions using six determinations per concentration, showed an inter-assay precision between 6.0% and 9.3% and an inter-assay accuracy between − 0.9% and 3.6%.

In the second aliquote TGA, Quick’s PT and APTT were measured before (baseline and DABI) and after the addition of idarucizumab (baseline + I and DABI + I). TGA was measured with the Technothrombin TGA RC Low reagent and substrate (Technoclone, Austria). Lag Time, Peak Thrombin, Time to Peak Thrombin (TPT), and Area Under the Curve referred to as Endogenous Thrombin Potential (ETP) were recorded. PT was measured using Thromborel S and APTT using Pathromtin SL (both Siemens Healthcare Diagnostics, Germany) on the CS-2500 coagulation analyzer (Sysmex, Kobe, Japan). PT results were expressed in percent and APTT in ratio to normal pool plasma obtained from 20 apparently healthy subjects. In samples containing idarucizumab (baseline + I and DABI + I) thrombin time (TT) was measured with the Test-Thrombin Reagent (Siemens Healthcare Diagnostics, Germany) on the same coagulation analyzer.

Statistical analysis was performed with GraphPad Prism 5.0 (GraphPad Software, USA). Average ± standard deviation (SD) was calculated for all the variates. Paired t-test was calculated between baseline and baseline + I samples and between DABI and DABI + I samples. When more than two groups were compared (baseline, baseline + I and DABI + I) ANOVA was assessed with the Tukey's multiple comparison post-hoc test. Pearson’s correlation coefficients were calculated between dabigatran concentration and coagulation assays. A p value < 0.05 was considered as statistically significant.

## Supplementary Information

Supplementary Information.
