# Supplementary material for: The in vitro addition of idarucizumab to plasma samples from patients increases thrombin generation
Source: Sci Rep. 2021 Mar 15;11:5920. doi: 10.1038/s41598-021-85318-y (PMC7961037; doi:10.1038/s41598-021-85318-y)
Supplement: Supplementary file 1 — Supplementary Information. [file 41598_2021_85318_MOESM1_ESM.docx]

**THE *IN VITRO* ADDITION OF IDARUCIZUMAB TO PLASMA SAMPLES FROM PATIENTS INCREASES THROMBIN GENERATION**

Mojca Božič Mijovski^1,5^, Rickard E. Malmström^2^, Nina Vene^1^, Jovan P. Antovic^3^, Alenka Mavri^1,4^

Supplement Table 1. TGA and screening coagulation tests before and after the addition of saline solution to plasma from patients who did not receive anticoagulant therapy. Average ± SD and paired t-test p values are shown.

|  | **Plasma**  (n = 8) | **Plasma + Saline solution**  (n = 8) | **p** |
| --- | --- | --- | --- |
| Thrombin generation |  |  |  |
| Lag Time (min) | 7.2 ± 1.0 | 7.2 ± 1.0 | NS |
| Peak Thrombin (nM) | 504 ± 104 | 503 ± 97 | NS |
| TPT (min) | 11.2 ± 1.9 | 11.3 ± 1.9 | NS |
| ETP (nM x min) | 4761 ± 636 | 4924 ± 621 | <0.001 |
| PT (%) | 99 ± 9 | 98 ± 9 | NS |
| APTT (ratio) | 1.03 ± 0.12 | 1.02 ± 0.11 | NS |

APTT – Activated Partial Thromboplastin Time, ETP – Endogenous Thrombin Potential, NS – not significant, PT – Prothrombin Time, TGA – Thrombin Generation Assay, TPT – Time to Peak Thrombin
